# Open science policy guidelines promoting open data sharing in low and middle-income countries for respiratory health research under NIHR Global RESPIRE project

**DOI:** 10.7189/jogh.15.03021

**Published:** 2025-07-01

**Authors:** Tapas Kumar Mohanty, Simon Smith, Tathagata Bhattacharjee, Christopher J Weir, John Norrie, RESPIRE Collaboration

**Affiliations:** 1 KEM Hospital Research Centre, Vadu Rural Health Program, Pune, India; 2 The University of Edinburgh, NIHR Global Health Research Unit on Respiratory Health (RESPIRE), Edinburgh, UK; 3 London School of Hygiene & Tropical Medicine, Department of Population Health, London, UK; 4 The University of Edinburgh, Edinburgh Clinical Trials Unit, Usher Institute, Edinburgh, UK; 5 Queen's University Belfast, Department of Social Science Research, Northern Ireland, UK

## Abstract

Open science drives progress, especially in the low-and-middle-income countries (LMICs), where data security and confidentiality are at risk due to lack of resources and non-compliance with diverse privacy laws. The National Institutes of Health and Care Research (NIHR) Global Health Research Unit on Respiratory Health (RESPIRE 2) project, funded by the UK NIHR, is a global collaboration led by the University of Edinburgh and Universiti Malaya, in partnership with seven LMICs and the UK. The collaboration developed open science policy guidelines to streamline data sharing, while ensuring compliance with privacy laws. They thus enable open data sharing in RESPIRE, furthering knowledge and scientific progress and providing greater research opportunities. This is in alignment with UNESCO, which promotes the open science movement to make scientific research and data more accessible, transparent, and collaborative. Some of the key components of this policy guideline are: Here we outline some of the key components of this policy guideline and provide recommendations. By following ethical data-sharing practices and fostering international collaboration, researchers, research assistants, technicians, and research support services can improve the impact of their research and contribute significantly to resolving global health challenges. Policymakers, research institutions, and funding agencies must support the adoption of open science practices in local contexts for long-term sustainability.

This policy brief summarises the Policy Guidelines on Open Science & Open Data: Envisioning Open Science Road Map for RESPIRE Project partner sites such as Bangladesh, Bhutan, India, Indonesia, Malaysia, Pakistan, Sri Lanka, and the United Kingdom. It was developed by the National Institute for Health and Care Research (NIHR) Global Health Research Unit on Respiratory Health (RESPIRE 2) project. This was prepared by the RESPIRE Supporting Platform III – ‘Open Science, Data, and Methodologies’, which focusses on the open science ambitions to promote Findable, Accessible, Interoperable, and Reusable (FAIR) principles for data sharing, thus encouraging data management planning, the use of standardised metadata, CC BY licensing, and so on [1]. This brief also encapsulates the data privacy regulations of the RESPIRE partner countries in the context of the seven Southeast Asian countries (Bangladesh, Bhutan, India, Indonesia, Malaysia, Pakistan, and Sri Lanka), along with the UK General Data Protection Regulation (GDPR) (**Box 1**).

Box 1 **Promoting open science practices.**

- FAIR data.
- Adherence to relevant legal and ethical frameworks.
- Compliance with all relevant data protection legislation.
- Informed consent for data preservation and sharing.
- Use of suitable data repositories, metadata standards, data management plans, and appropriate licenses (*e.g.* CC BY).

## BACKGROUND

Open science is essential for advancing scientific progress, especially in low-and middle-income countries (LMICs).

The lack of a secure infrastructure setting (*i.e.* a trusted research environment) in LMICs, compounded by funding constraints and insufficient training, often compromises data privacy and confidentiality. Moreover, these challenges are coupled by difficulty in complying with a variety of data privacy frameworks, including national data protection regulations, cross-border data sharing standards, and local institutional policies, as well as by geopolitical tensions, and the complexities inherent in working with vulnerable populations (*e.g.* working with the data of people in the refugee camps, tribal populations/indigenous populations, and so on). The RESPIRE Open Science Policy Guidelines address these barriers by providing a structured approach to data sharing, while adhering to local and international privacy regulations. This approach ensures that researchers in the LMICs can maximise research visibility and reproducibility (*e.g*. by making research data FAIR) and benefit from global open science initiatives, while continuing to ensure data privacy.

### Stakeholder involvement in developing these Policy Guidelines

Stakeholder input has played a crucial role in the formulation of these Policy Guidelines, from data sharing in RESPIRE phase 1 to the comprehensive open science policy and practices of phase 2. This includes mandating data management plans (DMPs) for all research partners, adopting and promoting FAIR data principles, and implementing secure data-sharing protocols. Stakeholders have also helped immeasurably by ensuring that global open science principles and the ethical, legal, and regulatory frameworks of partner countries are complied with.

Regular partner consultations, review meetings, and knowledge-sharing sessions were held, including conferences, webinars, and panel discussions. These facilitated the integration of diverse perspectives, particularly on ethical and legal matters. Stakeholders further assisted in the development of best practices for the use of secure repositories, data-sharing agreements, and efficient data transfer, and they also provided the impetus to begin building a network of data champions who would support local policy implementation and capacity building within research groups.

However, these Policy Guidelines are not directed solely at RESPIRE research partners: they are intended for all those associated with or engaging with stakeholders and researchers of the RESPIRE project.

## BENEFITS OF OPEN SCIENCE FOR LMICS

The United Nations Educational, Scientific and Cultural Organisation (UNESCO) plays a leading role in promoting open science. It is a movement to make scientific research and data more accessible, transparent, and collaborative. Per UNESCO recommendations, open data – data that is available without restrictions – is a key component of open science [2].

These Policy Guidelines promote open data sharing within the RESPIRE Projects, aligning with open science principles. Benefits of data sharing include advancing knowledge and scientific progress and providing greater opportunities to university and partner-country researchers to build on existing data/research. By making research data accessible, the project will gain several advantages:

- Increased visibility: making research data accessible boosts the visibility of scientific work, enabling it to reach a wider audience, including other researchers and policymakers. This can also improve the number of citations for published papers [3,4].
- Collaboration across borders: open data facilitates international collaboration, enabling LMICs to contribute to and benefit from global research networks. Researchers can build on one another’s work, thereby fostering innovation.
- Research efficiency: open access to existing data reduces redundancy in research efforts and accelerates discoveries by allowing researchers to build on previous studies without duplicating work, thereby saving time and money.
- Reproducibility of research: by ensuring data transparency, other researchers can verify findings and replicate studies, increasing the reliability of scientific outcomes and fostering public trust in academic research.

## KEY COMPONENTS

The first key component of these policy guidelines are FAIR principles for data sharing, which ensure that researchers can easily discover and reuse data, promoting collaboration across borders (Table S2 in the **Online Supplementary Document**). The second is balancing openness with data privacy. Legal compliance with partner countries’ data protection legislation and the UK GDPR is essential. Anonymisation should be employed to protect participants and their data while still making potentially valuable datasets accessible.

The key drivers for balancing openness with ethical data management and sharing activities are (Figure S2 in the **Online Supplementary Document**):

- verification of findings, ensuring research accuracy and integrity;
- replication of the study, supporting transparency and reproducibility;
- reuse of the data, enabling new analyses, collaborations, and broader scientific impact.
- open data sharing, while it is beneficial, it poses risks of potential data misuse.

To mitigate these risks:

- All shared data must be anonymised to protect the participants’ privacy; data should also be screened to prevent sharing of indirect identifiers.
- Active data should be shared with appropriate access control measures, limiting access to authorised personnel only.
- Personnel involved in the study should sign data use agreements encompassing the uses of data and the consequences of misuse.
- Regular audits should be conducted to ensure data accuracy data usage and compliance with ethical standards.

The third key component relates to ethics and legal frameworks. Transparency and integrity are essential in this context. Research participants, the general public, and government organisations must be able to see and understand how research data is collected, processed, preserved, and shared.

To raise awareness of all partner countries’ legal requirements and support legal compliance, an overview of Data Protection principles and provisions was added to the Policy Guidelines. Every research partner that is involved in RESPIRE 2 must adhere to all relevant data protection legislation in their country.

### Bangladesh

The provisions of data protection and privacy are laid out by the rights of privacy enacted under Point no. 43, The Constitution of the People’s Republic of Bangladesh, Point 43 of the 2006 Information Communication Technology Act [5], and the 2018 Digital Security Act [6]. The latter defines terms such as ‘digital’, ‘digital device’, ‘digital security’, ‘data storage’, and ‘critical information infrastructure’, among others.

Under the provisions of the Digital Security Act of Bangladesh, the National Digital Security Council is empowered to formulate and issue data protection guidance as and when required, while, the Digital Security Agency is authorised to handle executive functions, such as blocking content or decrypting a data source. The Act includes provisions to ensure the protection of critical digital infrastructure and addresses crimes such as unauthorised data access, identity fraud, and cyber terrorism, while also safeguarding rights such as the freedom of information as outlined under the 2009 Right to Information Act [7].

### Bhutan

The 2023 National Digital Identity Act of Bhutan [8] governs the framework for the protection of personal data. It mandates adherence to globally recognised standards for data security and privacy, holding all entities (issuers, verifiers, and trust service providers) accountable for safeguarding data secrecy (Sections 61 and 63). Specific guidelines regulate data usage, access, and disclosure and require conformance to trust policies and audits (Sections 27, 63). The Act also enforces strict data residency within Bhutan, with limited exceptions under controlled conditions (Sections 115–117). These provisions collectively aim to ensure robust protection and integrity of personal information. In addition, the 2018 Information, Communications Technology, and Media Act [9]is the primary legislation governing the information and communication technology and media sector in Bhutan. The Act has provisions for the protection of users' data and privacy, and also provides for the rights of individuals, including the right to access their data, the right to correct inaccurate data, the right to object to the processing of their data, and the right to have their data erased. However, the Governance Framework of the National Digital Identity Act takes precedence and can set rules for storing, managing, or transferring digital identity information outside Bhutan when it involves entities within the National Digital Identity Infrastructure.

### India

The 2023 Digital Personal Data Protection Act [10] and the 2000 Information Technology Act [11] are the two primary laws governing the privacy and protection of personal data in India. It regulates the collection, processing, and use of personal data by government agencies, including research institutions, and private companies in India. It describes the principles that must be followed by organisations collecting, processing, or storing personal data, and also ensures that individuals have control over their personal data and that their data is used responsibly and ethically. The Data Protection Board of India will be formed to adjudicate non-compliance with the provisions of the Act.

### Indonesia

The 2022 Data Protection Law of Indonesia is a comprehensive law regulating personal data processing in Indonesia [12]. It applies to all organisations that process personal data (both electronic and non-electronic means) in Indonesia, regardless of their size or location. It establishes the Indonesian Data Protection Authority and its roles and responsibilities (Articles 58–61). The regulation empowers individuals with several key rights, such as the confidentiality of personal data, the right to file complaints regarding data breaches, the right to access and correct data without disrupting the system, the right to obtain data usage history, and the right to request data deletion, unless restricted by law.

### Malaysia

The 2010 Personal Data Protection Act of Malaysia [13] encapsulates the regulations to protect personal data emphasising the fair and lawful processing of personal data, requiring informed consent and notification of data usage purposes. It also established the Personal Data Protection Commission to oversee compliance (Section 47, p. 54). The Act allows law enforcement and regulatory authorities to access personal data under certain conditions, such as compliance with legal obligations or for the administration of justice (Section 6(2), p. 18).

### Pakistan

The Constitution of the Islamic Republic of Pakistan guarantees the privacy of the home alongside the dignity of every man and woman as their fundamental right under Article 14. The draft Personal Data Protection Bill [14] has not yet been enacted at the time of writing. It aims to provide individuals with control over their personal data. It also establishes the Pakistan Data Protection Authority to oversee compliance with the act. The same provision only allows personal data to be transferred outside of Pakistan when that other country offers at least the same level of personal data protection.

### Sri Lanka

The 2022 Personal Data Protection Act, no. 9 (p. 1, preamble) [15] provides the comprehensive framework that regulates the processing of personal data. The Act strengthens the rights of data subjects by mandating that the controllers process personal data in a lawful, fair, and transparent manner (Sections 4–5, p. 3–5). In addition, the legislation provides for the establishment of the Data Protection Authority.

### UK

The 2018 General Data Protection Regulation [16] covers the processing of all Personal and Special Category data, which includes all operations performed upon the data, from collection to disposal.

## POLICY RECOMMENDATIONS

### Data management plans

Ensure that all researchers involved in the RESPIRE project draft and implement DMPs and apply to consistent and metadata standards to improve data reuse and interoperability. Each dataset should be accompanied by a readme file: a plain text (.txt) file that provides the following information (Figure S3 in the **Online Supplementary Document**):

- collection methods and tools used (*e.g.* hardware, software, instruments);
- standards and calibrations applied during data collection;
- variables captured in the dataset;
- file and document formatting details;
- file naming and versioning conventions to track data changes;
- definitions, field codes, labels, symbols, and abbreviations for clarity;
- processing information, including anonymisation procedures and quality assurance steps;
- analysis methods used to process or interpret the data;
- outliers identified and how they were handled.

### Quality assurance

To ensure data accuracy and consistency, quality assurance processes must be implemented for all data processing activities such as collection, data entry, transcription, anonymisation, and analysis. They should be documented in the project’s data management plan, alongside standard operating procedures governing data quality checks.

### Adopt open licensing

FAIR data should be licensed to facilitate its reuse and ensure that researchers worldwide can build upon it. Promoting the use of open licenses such as Creative Commons (*e.g.* CC BY) to maximise data reuse while ensuring proper attribution to data creators.

### Strengthen data privacy safeguards

To comply with national and international legal requirements, research data should be de-identified and stored in secure (preferably networked) storage systems. Research participants must be informed about how their data will be used and safeguarded.

### Provide training and capacity building

Organise and run regular workshops to educate researchers about ethical data-sharing practices and the requirements of relevant data protection laws. Ensuring that researchers are aware of their legal obligations will help mitigate any potential violations.

### Data sharing

In line with the University of Edinburgh’s Research Data Management Policy [17] and RESPIRE Data Management Guidance, RESPIRE 2 has adopted a FAIR approach to data sharing. All the research partners must ensure that the de-identified data from their respective studies is deposited in an open access repository, such as Edinburgh DataShare [18]. This will ensure the long-term sustainability of the data, while making it accessible to a global audience. In addition, our future work will explore federated data-sharing approaches to accommodate data owners' preferences and legal requirements, thereby ensuring flexibility and compliance.

### Informed consent

Informed consent is essential if ethical standards are to be maintained and legal requirements met. When obtaining consent from their participants, researchers should clearly explain the process and purpose of data sharing. Adopting a simple three-step communications strategy will make this process easier:

- Step 1: define your terms, *i.e.* clearly explain key terms such as data sharing, and data preservation, and their implications.
- Step 2: give reasons, *i.e.* provide rationale for data sharing, including benefits, such as verification, reproducibility, and reuse of data.
- Step 3: highlight the advantages of data sharing, such as ensuring research integrity, transparency, and faster progress by reusing the data.

As stated in the University of Edinburgh Research Data Management Policy [17], research data should be offered for deposit in a suitable national or international data service, disciplinary repository, or institutional repository.

The University of Edinburgh provides the free-at-point-of-use, open-access digital repository: the Edinburgh DataShare [18]. DataShare is a trusted digital repository, awarded with the CoreTrustSeal [19]. It is optimised for search engines and indexed in Google Scholar and Google Dataset to promote data discovery.

For RESPIRE 2 research partners, depositing data in DataShare is more complicated than it would be for University of Edinburgh staff. The process will involve transferring the data to a member of the project team based in Edinburgh who will then deposit the data on behalf of the research partner. This deposit process will be supported by the University of Edinburgh Research Data support team.

### Specific challenges for LMICs and mitigation strategies

The LMICs are at a junction where progress and geopolitics meet. They consequently face a diverse range of regulatory norms, which reflect the typical regional problems. In particular, there are three major challenges to LMICs wishing to adopt open science in practice.

The first is technical, including limited digital infrastructure, unstable internet access, and lack of secure data storage. Investing in cost-effective cloud or networked servers, using offline data capturing tools like Research Electronic Data Capture (REDCap), and exploring the plausibility of establishing the decentralised, federated research data repository are recommended.

The second challenge is cost and resource constraints such as limited funding, researcher training costs, high-cost factors of publication, procuring devices/tools, and maintaining data-sharing platforms. Leveraging public-private partnerships; information, education, and communication/behaviour change communication activities; making open science the new normal; and encouraging the use of free and open-source software and tools such as the Open Science Framework (OSF) can help mitigate these problems.

The third is the diverse ethical and regulatory norms of regional contexts which are major barriers to a standardised data governance framework. This is particularly important when working with the data of potentially vulnerable populations, such as those in refugee camps and with tribal populations. The strategies to overcome this third challenge include data-sharing agreements, data management plans, and safety protocols, which align with relevant data protection legislation.

Communication and engagement are equally important, particularly when working with vulnerable and tribal communities. Researchers must be encouraged to engage with research participants and their communities, clearly articulating the public health benefits of FAIR data sharing. In addition, drawing on the Collective Authority, Authority to Control, Responsibility, and Ethics (CARE) principles can be a valuable tool when working with vulnerable communities and tribal populations. CARE emphasises engagement with research participants and their communities, clearly articulating the public health benefits of FAIR data sharing [19].

### Monitoring policy implementation, compliance, and impact

Periodic consultations with RESPIRE Partners and collaborators as well as cross-cutting activities with other RESPIRE supporting platforms (*e.g.* capacity building, stakeholder engagement, regular updates on emerging technologies and digital health; see Policy Guidelines on Open Science & Open Data, Version 1, November 2023, pp. 1–5) have functioned as the policy adoption interface. The policy adoption interface brings together the reflections from RESPIRE 1 and RESPIRE 2 (to date) to guide the adoption of open science practices. Given the dynamic nature of this work, monitoring policy implementation and compliance progresses incrementally. The implementation process is supported through the development of open science policy guidelines, capacity-building initiatives, and the identification of context-specific good practices aiming at the best practices.

As noted above, compliance with regional regulations and legal frameworks is ensured by frequent partner consultations, which promote FAIR principles, and the mandating of data protection impact assessments (a structured process designed to identify, assess, and mitigate risks associated with data processing) and DMPs. This has enabled partners to obtain necessary regulatory approvals. Further, local data champions play an essential role in addressing local regulatory challenges, internal compliance monitoring, and adjusting strategies to align them with the evolving policies and technologies in partner countries.

**Figure 1** represents a conceptual framework for monitoring the policy adoption, compliance, and impact. The Open Science Policy can be implemented with three core themes (the center triangle – each edge represents a core pillar of open science policy adoption): ensuring FAIR and CARE principles; adhering to data governance & ethical oversight; and collaborative research. Within the cross-cutting domains (triangle edges), each edge represents a cross-cutting pillar such as:

- FAIR & CARE principles and data governance, and ethical oversight: governance and compliance (top section of the tringle) – help the researcher know more about the privacy and regulatory norms and their compliances, motivating them for responsible practice of open science where transparency, sharing, and openness happen with clear boundaries of respect, privacy, and legal compliance.
- Data governance & ethical oversight and collaboration: data management and impact assessment (right section of the tringle) helps in standardising the DMPs, along with periodic open science policy review and internal data monitoring.
- FAIR & CARE principle and collaboration: capacity building and stakeholder engagement (left section of the tringle) – the researchers were trained, learning about their role and working with the local data champions for better communication and implementation.

The base and the two sides of the outer tringle represents the overarching enablers that encapsulate the entire open science policy implementation process:

- Innovation & sustainability (base of the outer triangle): provides the foundation for long-term sustainability by adapting strategies such as public private partnership, decentralised federated data infrastructure, and capacity building.
- The two sides – feedback mechanism and monitoring & evaluation matrix: they act as active pathways for iterative learning from experience and tracking progress. Together, these overarching elements help ensure that it can be easily implemented and adopted envisioning a long-term impact.

## CONCLUSIONS

Open science offers LMICs a unique opportunity to advance respiratory health research. By following ethical data-sharing practices and fostering international collaboration, researchers, research assistants, technicians, and research support services can improve the impact of their research and contribute significantly to the tackling of global health challenges.

### Call to action

Policymakers, research institutions, and funding agencies must actively support the adoption of open science practices. The following strategies and steps can be adapted to suit the local research context thereby ensuring long-term sustainability:

- Active data should be shared with appropriate access control measures, limiting access to authorised personnel only.
- Establish and strengthen Public-private partnerships (PPPs) to mobilise funding and technical support across government bodies, research institutions, and private sectors.
- Providing resources for data-sharing platforms through capacity building and local expertise, while offering technological solutions such as secure cloud storage solutions and improved internet connectivity.
- Implementing standardised data management policies that mandate open science practices and the use of DMPs.
- Ensuring data privacy and legal compliance, including adherence to national regulations and across-border data protection requirements.
- Promoting international research collaborations to enhance knowledge exchange, build networks, and access diverse funding opportunities.
- Adopting federated data-sharing platforms to allow institutions to retain control of their active datasets while enabling interoperability with global research collaborators.
- Introducing membership-based or cost-sharing models to support the ongoing maintenance and sustainability of data-sharing infrastructure.

Keeping the above strategies in mind, stakeholders can take meaningful steps to improve data-sharing efficiency, foster collaboration, and enhance research impact within and beyond LMIC settings (**Table 1**).

**Table 1:** Actionable steps for enhanced data-sharing efficiency and accessibility

	Description
**Step 1**	Identify regional data-sharing hubs within the selected research centres or universities that already have the necessary infrastructure, thereby significantly reducing capital expenditure and setup time.
**Step 2**	Establish standard data governance frameworks and standard safety protocols on privacy and ethical considerations.
**Step 3**	Facilitate stakeholder training and engagement by conducting workshops on capacity building involving researchers, policymakers, and data managers.
**Step 4**	Encourage the use of cost-effective open-source repositories and free open-source tools to reduce the operational costs.
**Step 5**	Implement a monitoring and evaluation framework to assess data accessibility and to provide open science monitoring.

## Additional material


Online Supplementary Document

**Figure 1. **Conceptual framework for monitoring the policy adoption, compliance and impact.
