# Supplementary material for: Open science policy guidelines promoting open data sharing in low and middle-income countries for respiratory health research under NIHR Global RESPIRE project
Source: J Glob Health. 2025 Jul 1;15:03021. doi: 10.7189/jogh.15.03021 (PMC12208280; doi:10.7189/jogh.15.03021)
Supplement: Online Supplementary Document [file jogh-15-03021-s001.pdf]

# JOURNAL OF GLOBAL HEALTH – SUPPLEMENT

**Supplement to: Mohanty TK, Smith S, Bhattacharjee T, Weir CJ, Norrie J; RESPIRE Collaboration. Open science policy guidelines promoting open data sharing in low and middle-income countries for respiratory health research under NIHR Global RESPIRE project. J Glob Health. 2025;15:03021.**

**Figure S1.** Benefits of Open Science

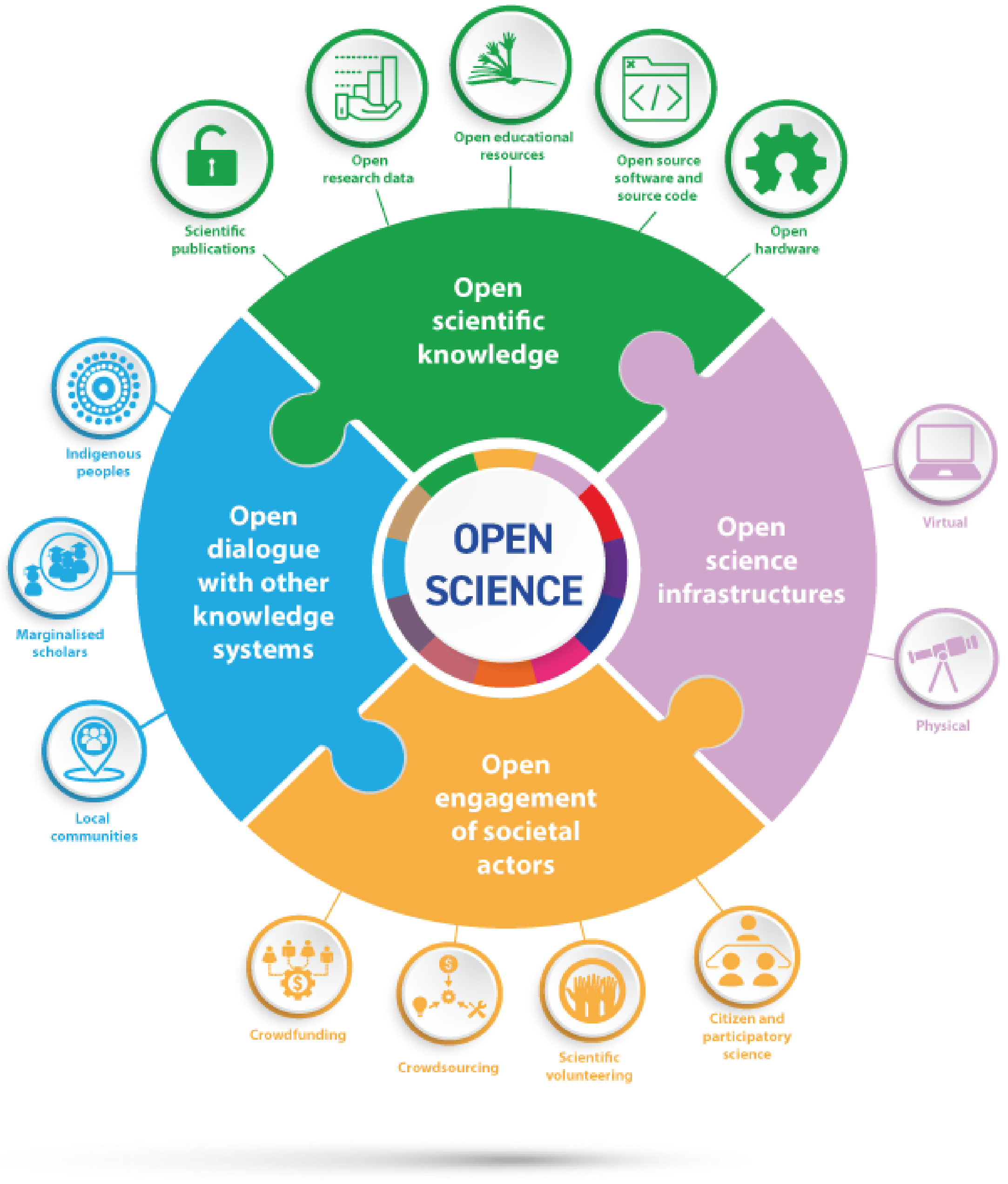


Open Science domains and actors adapted from the UNESCO Recommendation on Open Science (2021) licensed under CC BY-SA 4.0

**Figure S2.** Key Drivers of Research Data Management(RDM) and Data Sharing

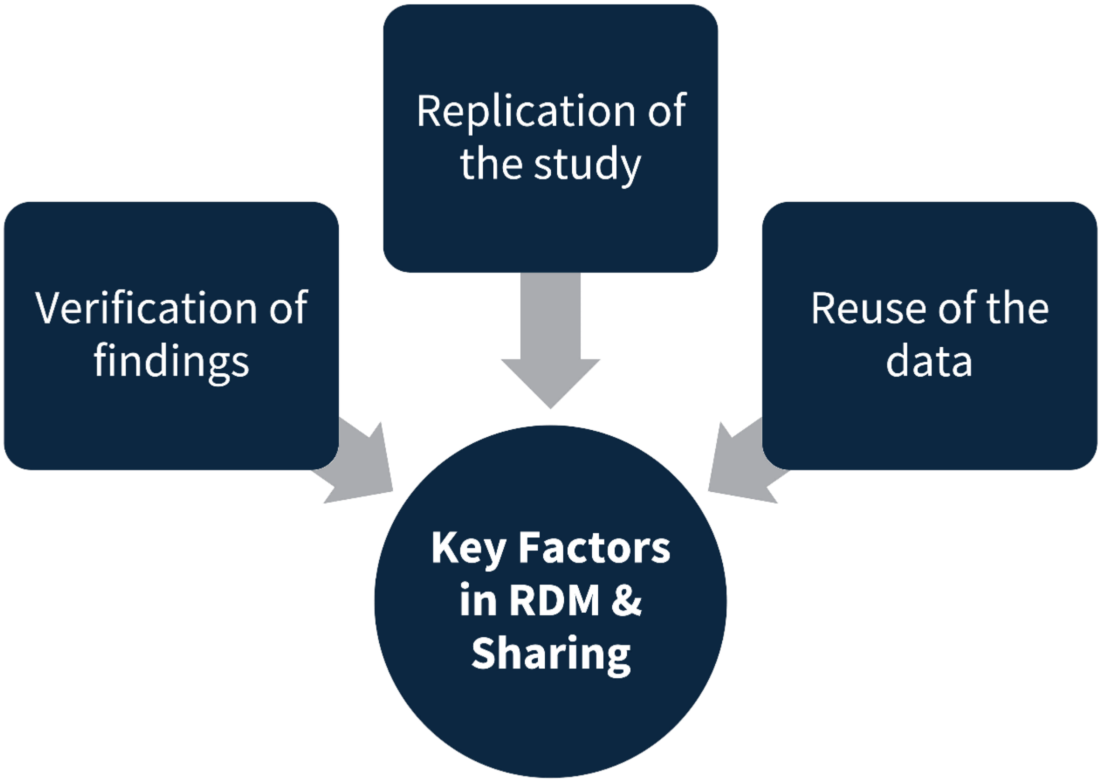

**Figure S3.** Readme style documentation

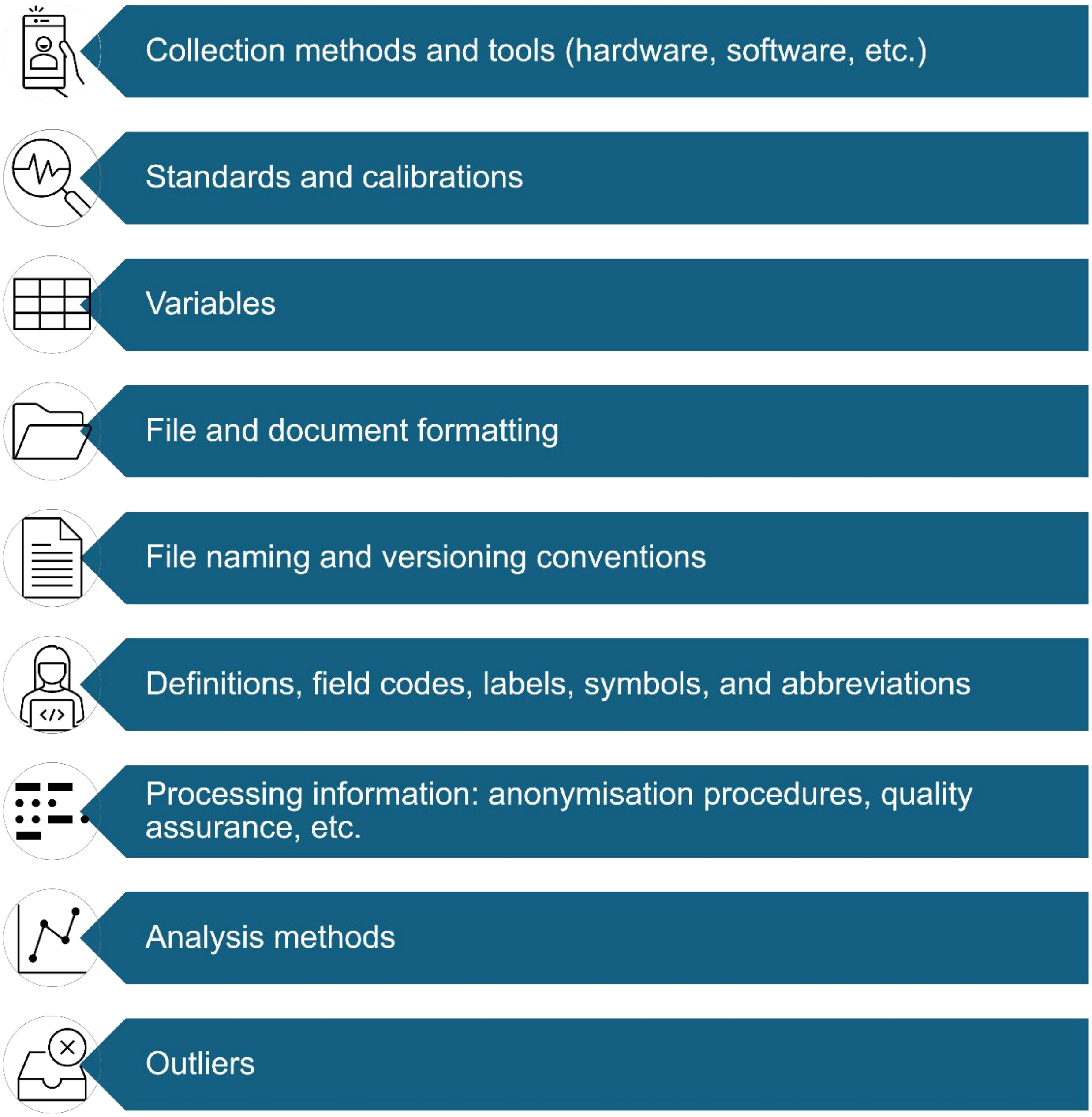

**Figure S4.** Three-step communication strategy to ensure informed consent

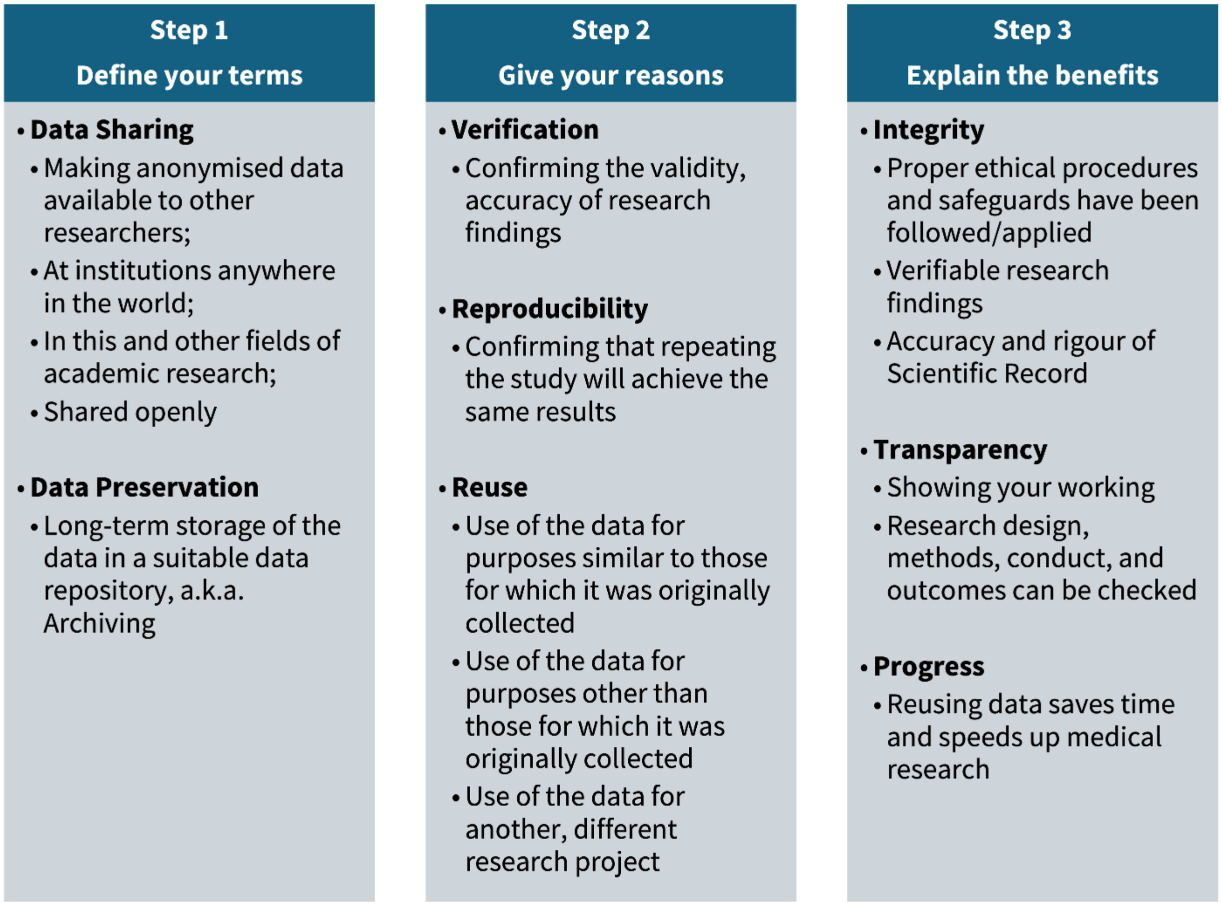


**Table S2.** FAIR Principles

| Findable | Accessible | Interoperable | Reusable |
|---|---|---|---|
| archived in a repository, has good metadata and keywords, assigned a persistent identifier (e.g., a DOI*), publication of findings includes a Data Availability Statement | access conditions/restrictions are clear, good documentation, accessible data formats | uses open/standard file formats, metadata are machine-readable, clearly defined/standard ontologies | has good documentation and metadata, is licensed (CC BY 4.0)†, is citable |

*DOI – Digital Object Identifier
†CC BY 4.0 – Creative Commons Attribution
